# Altered distribution of resting periods of daily locomotor activity in patients with delayed sleep phase disorder

**DOI:** 10.3389/fpsyt.2022.933690

**Published:** 2022-10-13

**Authors:** Marina Hirose, Toru Nakamura, Akiko Watanabe, Yuichi Esaki, Shigefumi Koike, Yoshiharu Yamamoto, Nakao Iwata, Tsuyoshi Kitajima

**Affiliations:** ^1^Department of Psychiatry, Fujita Health University School of Medicine, Toyoake, Japan; ^2^Graduate School of Engineering Science, Osaka University, Osaka, Japan; ^3^Department of Sleep Medicine, Toyohashi Mates Sleep Disorders Center, Toyohashi, Japan; ^4^Educational Physiology Laboratory, Graduate School of Education, University of Tokyo, Tokyo, Japan

**Keywords:** circadian rhythm sleep disorders, delayed sleep phase disorder, behavioral organization, depression, actigraphy

## Abstract

Delayed sleep phase disorder (DSPD) and mood disorders have a close relationship. However, the shared mechanisms by DSPD and mood disorders have not been well-elucidated. We previously found that micro-fluctuations in human behaviors are organized by robust statistical laws (behavioral organization), where the cumulative distributions of resting and active period durations take a power-law distribution form and a stretched exponential functional form, respectively. Further, we found that the scaling exponents of resting period distributions significantly decreased in major depressive disorder (MDD). In this study, we hypothesized that DSPD had similar characteristics of the altered behavioral organization to that of MDD. Locomotor activity data were acquired for more than 1 week from 17 patients with DSPD and 17 age- and gender-matched healthy participants using actigraphy. We analyzed the cumulative distributions of resting and active period durations in locomotor activity data and subsequently derived fitting parameters of those distributions. Similar to patients with MDD, we found that resting period distributions took a power-law form over the range of 2–100 min, with significantly lower values of scaling exponents γ in patients with DSPD compared with healthy participants. The shared alteration in γ suggests the existence of similar pathophysiology between DSPD and MDD.

## Introduction

Circadian rhythm sleep disorders (CRSD) are disabling clinical disorders that prevent patients from entraining their own circadian sleep–wake rhythm with their environment or social light–dark cycles (1). In particular, patients with delayed sleep phase disorder (DSPD), a representative type of CRSD, exhibit significant delays in sleep onset time and awakening time and have difficulty sleeping at a desired time; however, once they fall asleep, they can basically continue sleeping with enough quality and duration (1).

Several hypotheses have been proposed to explain the pathophysiology of DSPD, including a longer circadian period, changes in resetting circadian rhythms in response to light stimulus, clock gene polymorphisms, changes in sleep homeostasis, and behavioral tendencies (2); however, the precise mechanisms responsible for DSPD remain unknown (3). One key issue for discussion may be the close relationship between DSPD and mood disorders. Mood disorders or depressive symptoms are often observed in patients with DSPD (1, 4, 5). Conversely, circadian disturbance and/or evening chronotype is common in patients with mood disorders (6–8), and are associated with an increased risk of mood episode relapse (9, 10). Chronobiological interventions, such as light therapy, have therapeutic properties for both DSPD and mood disorders (1, 7). Many reports have also reported associations between the circadian regulatory system, including clock genes, and mood regulation (11, 12), thus suggesting the existence of some shared mechanisms by DSPD and mood disorders.

As a further chronobiological insight, our previous studies have reported that micro-fluctuations in daily life locomotor activities measured by actigraphy are organized by robust statistical laws (referred to as behavioral organization), with resting and active period durations on ultradian time scales following a power-law and a stretched exponential cumulative distribution, respectively (13, 14). Furthermore, we discovered significantly lower parameter values (power-law scaling exponents) for resting period durations, indicating a systematic increase in the incidence of longer resting periods in patients with major depressive disorder (MDD) compared to controls (13, 14). Interestingly, this characteristics were also observed in the rest–activity of mice with mutation in a circadian clock gene, *Period2* (13). In the present study, we hypothesized that patients with DSPD also show a similar alteration in the statistical laws of behavioral organization, thus examined the distributions of resting and active period durations in locomotor activity in daily life.

## Materials and methods

### Participants

Between September 2013 and June 2014, 22 patients with DSPD were treated at the Department of Psychiatry, Fujita Health University Hospital, or the Department of Sleep Medicine, Toyohashi Mates Sleep Center. Inclusion criteria for patients were as follows: diagnosis of DSPD according to the diagnostic criteria of the International Classification of Sleep Disorders, Second Edition (ICSD-2) (15) and confirmed by sleep physicians (certified by the Japanese Society of Sleep Research), an allowed range of age was between 12 and 65 years old. Enrolled patients included both of those with and without improvement after treatment. DSPD was renamed delayed sleep–wake phase disorder (DSWPD) in the ICSD-3 (16), and all patients also met the DSWPD criteria. Patients with a comorbidity of other psychiatric disorders (e.g., mood disorders including MDD and bipolar disorder, anxiety disorder, schizophrenia, and developmental disorders) were intentionally excluded from the study.

Age- and gender-matched twenty-two healthy participants having no apparent psychiatric disorder or disturbance of sleep–wake rhythm, confirmed by physicians, were selected as control participants. Their data were collected almost contemporaneously with those of the patients. This study was approved by the Ethics Committee of the Fujita Health University and Department of Sleep Medicine, Toyohashi Mates Sleep Center. All participants (patients and control participants) provided prior verbal and written informed consent. In the case of minors, consent was provided by the patient and one parent.

### Data collection

After providing informed consent, participants were given several questionnaires: Beck Depression Inventory (BDI), Morningness–Eveningness Questionnaire (MEQ), Severity Level Criteria for delayed sleep phase syndrome (DSPS; an alternate name for DSPD) (17), and our original questionnaire for CRSD symptoms (described below). Patients were instructed to maintain a sleep log and wear an actigraphy device (described below) for at least 1 week to collect locomotor activity data. We extracted related clinical information from medical records such as social adjustment (including whether the patient was on vacation/leave or not) and medication lists.

### Assessment of clinical symptoms

Subjective depressive state was determined using the BDI, a self-administered questionnaire devised by Beck et al. (18), that consists of 21 questions. The severity of depressive symptoms is represented by the total score (0–63 points). The temporal state of morningness–eveningness was assessed by MEQ, a well-established, self-administered questionnaire originally described by Horne and Ostberg (19), that consists of 19 questions. Of its total score (16–86 points), higher scores indicate morningness, whereas lower scores indicate eveningness. Severity Level Criteria for DSPS devised by Ando et al. (17) categorized the severity of DSPD according to sleep onset and offset times. Its detailed contents are described elsewhere (20). In brief, the severity of DSPD was assigned to one of four categories (“remission,” “mild,” “moderate,” or “severe”) based on a combined score of the extent of delaying of sleep onset and offset times. We also collected clinical symptoms of DSPD, including sleepiness, with our original questionnaire (21). Because this questionnaire has not yet been formally validated, we used the score of individual items required for the analysis. These clinical parameters were utilized as independent variables in analyzing the association with behavioral organization described below, where BDI and MEQ were used as continuous variables and Severity Level Criteria for DSPS were used as a categorical variable.

### Actigraphic assessment and analysis of rest–activity data

Locomotor activity data were collected using MicroMini RC actigraph (Ambulatory Monitors Inc., Ardsley, NY, USA), with a preset zero-crossing mode and 1-min recording epoch. The participants were instructed to wear it on the wrist of their non-dominant hand throughout the recording period for >7 days, unless the actigraph was likely to get wet (e.g., while bathing), or the participants were going to exercise strenuously. Activity data recorded as the device was being removed were identified and excluded from the analysis. We determined sleep parameters (times of sleep onset and offset, time in bed, total sleep time, sleep onset latency, latency to persistent sleep, wake after sleep onset, and sleep efficiency) based on the actigraphic data. Those parameters were automatically scored using the AW2 software (Ambulatory Monitoring, Inc., Ardsley, NY, USA), and were manually corrected, if required, by cross referencing the sleep logs.

**FIGURE 1:**
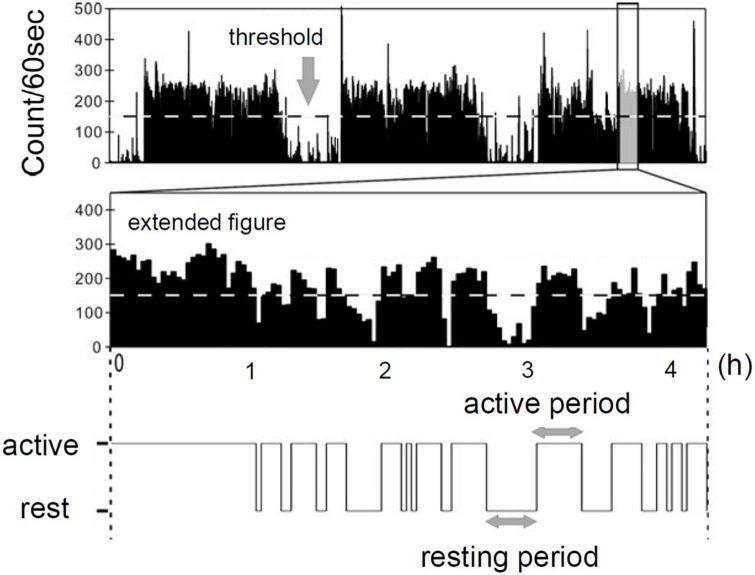
Definition of resting and active period by actigraph [Modified from Nakamura et al. (22) with permission].

The behavioral organization analysis that examines statistical laws of resting and active period durations in locomotor activity data was devised in our previous studies (13, 14). We first defined threshold as the average of non-zero activity counts (horizontal dotted line in Figure 1). The threshold was individually determined for each participant; therefore, it differed from participant to participant. The entire period of data was then binary categorized into active (continuously higher than the threshold) and resting (continuously lower than the threshold) periods (Figure 1; 22). We then estimated cumulative distributions *P*c(x≥*a*) of duration *a* (min) of both active and resting periods. These were calculated by numerically integrating the obtained probability density functions of both periods with a bin width of 1 min. We considered that cumulative distributions of active period durations take a stretched exponential functional form [*Pc*(*x*≥*a*) =*exp*(−α*a*^β^)], whereas, resting period durations follow a power-law distribution [*Pc*(*x*≥*a*) =*Ca*^−γ^] (13, 14). Here, the parameter γ is called scaling exponent of the distribution.

The parameters α and β of active period distributions were estimated by fitting a stretched exponential function to the estimated cumulative distributions based on the Levenberg–Marquardt algorithm (23). The scaling exponent γ of resting period distribution was determined using the maximum likelihood estimation approach proposed by Clauset et al. (24), which is a statistically robust method for quantifying power-law behavior in empirical data. We set the fitting range to [10, 100] min for cumulative distributions of active periods and *a* ≥ 3 min for resting periods. The distributions were analyzed separately for each participant, and the resulting values were used in the statistical analyses described in the next subsection.

### Statistical analysis

As a main outcome measure of this study, we tested the difference of parameter γ between healthy participants and patients with DSPD using an unpaired *t*-test. We also tested other statistical measures related to the distributions metrics (mean resting and active period durations, α, β) and other basic metrics (mean activity levels, standard deviation of locomotor activity, sleep onset time, and sleep offset time) using unpaired *t*-test to profile other behavioral characteristics of DSPD. When there was a significant difference in parameter γ between the groups, a multiple linear stepwise regression analysis was performed as an exploratory analysis with regard to the relationship between parameter γ as a dependent variable and the patient’s clinical parameters (MEQ, BDI, remission or non-remission, social adjustment, sleepiness, medications) as independent variables. In the latter analysis, we used the stepwise forward selection method with a threshold of *p* < 0.25. These clinical parameters were dichotomized for the analysis except for MEQ and BDI scores. Remission or non-remission corresponded, respectively, to “remission” or the other three categories in Severity Level Criteria for DSPS. Social adjustment was defined as whether they could adjust regularly to social schedules (job or school; vacation or long absence was excluded). Sleepiness was determined based on an item in our original questionnaire (21). The presence or absence of drugs with psychotropic or circadian properties was determined as for three drug categories separately (ramelteon, GABAergic agents such as benzodiazepines and non-benzodiazepine hypnotics, and vitamin B_12_). We used the JMP 8.0 software package (SAS Institute Japan, Tokyo, Japan) for all statistical analyses. A *p*-value of <0.05 was regarded as statistically significant.

## Results

### Demographics of patients

Of the 22 enrolled patients with DSPD, five patients did not wear the actigraph for a sufficient period of time. Therefore, the remaining data from 17 patients with DSPD (11 males and six females; mean age, 23.8 ± 11.0 years) and age- and gender-matched 17 healthy participants (11 males and six females; mean age, 23.6 ± 10.9 years) were analyzed in this study. Their demographics are summarized in Table 1. Two patients were not medicated; 15 patients took one or more medicines to aid their sleep during the study period as follows: ramelteon (*n* = 11), GABAergic agents [zolpidem (*n* = 3), zopiclone (*n* = 2), brotizolam (*n* = 2), clonazepam (*n* = 1), and tofisopam (*n* = 1)]; and vitamin B_12_ (*n* = 6) (with some overlap). Actigraphic data from 12.6 ± 5.9 days on average (range: 6.5–22.7 days) were used for the analyses, and no significant correlation between the number of days and the distribution parameter γ was observed (data not shown). The average proportion of the excluded data due to removal of the devise was 6.3 ± 5.0%.

**TABLE 1:** Patient characteristics.

	Patients (*n* = 17)	Controls (*n* = 17)	*P*-value
Age (years)	23.8 ± 11.0	23.6 ± 10.9	0.9505
Gender (Male/Female)	11/6	11/6	-
**Sleep parameters (actigraphy)**			
Sleep onset time (clock time)	1:56 ± 2:20	23:53 ± 0:53	0.0018*
Sleep offset time (clock time)	9:36 ± 2:12	7:01 ± 0:43	<0.0001*
Time in Bed (min)	473.2 ± 39.0	440.7 ± 39.8	0.0222*
^$^Total sleep time (min)	415.1 ± 59.4	404.6 ± 37.5	0.54
Sleep onset latancy (min)	6.7 ± 1.0	6.8 ± 1.1	0.89
Latancy to persistent sleep (min)	15.7 ± 10.3	10.0 ± 4.2	0.043*
Wake after sleep onset (min)	40.8 ± 31.0	23.8 ± 13.9	0.0472*
^#^Sleep efficiency (%)	87.5 ± 8.2	91.8 ± 3.0	0.0495*
Severity level criteria for DSPS	2.2 ± 2.1	0.7 ± 0.8	0.0091*
MEQ	41.2 ± 9.8	53.5 ± 7.2	0.0002*
BDI	8.6 ± 7.1	3.8 ± 2.7	0.0129*

MEQ, morning–eveningness questionnaire; BDI, Beck depression inventory.

*Statistically significant difference (*p* < 0.05).

^$^“Time in bed” was defined as the duration of the “DOWN” interval automatically analyzed using the AW2 software (Ambulatory Monitoring, Inc., Ardsley, NY, USA) and manually corrected if necessary.

^#^“Sleep efficiency” was defined as (total sleep time)/(time in bed)*100 [whereas, the AW2 software calculate “sleep efficiency” as (total sleep time)/(sleep period time)*100].

Both sleep onset time and sleep offset time in patients with DSPD were significantly delayed compared with those in control participants (sleep onset time delayed by 2.05 [h] on average, *p* = 0.0016; sleep offset time delayed by 2.51 [h], *p* < 0.0001), indicating the significant shift of circadian rhythms toward eveningness in patients with DSPD. Indeed, the group average score of Severity Level Criteria for DSPS in patients was significantly higher than that in control participants (DSPD: 2.2 ± 2.1, Control participants: 0.7 ± 0.8, *p* = 0.0091). Furthermore, the MEQ score in patients with DSPD was significantly lower than that in control participants (DSPD: 41.2 ± 9.8, Control participants: 53.5 ± 7.2, *p* = 0.0002). With respect to length and quality of sleep, patients with DSPD showd significantly longer time in bed longer latency to persistent sleep, longer wake after sleep onset, and lower sleep efficiency than did control participants. The BDI score was slightly but significantly higher in patients with DSPD compared with that in controls (DSPD: 8.6 ± 7.1, Control participants: 3.8 ± 2.7, *p* = 0.0129), although patients with DSPD had no apparent clinical depression (Table 1).

### Behavioral organization parameters in patients with delayed sleep phase disorder compared with those in the control participants

**FIGURE 2:**
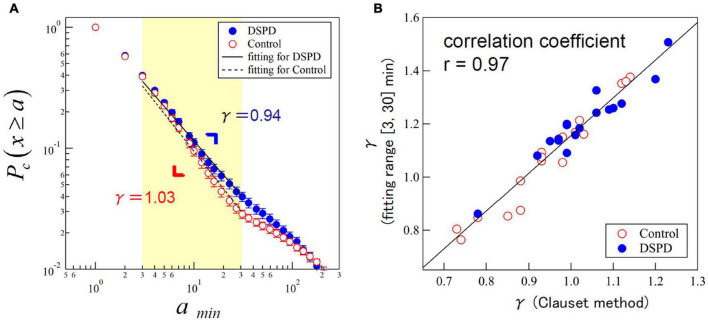
**(A)** Cumulative distributions of resting period durations (represented by parameter γ) in patients with delayed sleep phase disorder (DSPD) (blue circles) and control participants (red circles). The scatterplots present an average of the individual distributions and the error bars indicate the standard error of the mean. Straight lines are eye guides with overall mean values of γ = 0.94 for patients and γ = 1.03 for control participants. **(B)** Scatter plot of estimated values of γ by the Clauset method (x ≥ 3) versus the Levenberg–Marquardt algorithm with a fitting range of [3, 30] min.

The group average value of γ was significantly lower in the DSPD patients than in the control participants (DSPD: 0.94 ± 0.13, control participants: 1.03 ± 0.11, *p* = 0.0496) (Table 2). We examined the effects of resting distributions on the estimation of scaling exponents to confirm the robustness of our finding; the Clauset method assumes that the observed data follow power-law distribution over the scaling region *x*≥*xmin*, where *xmin* is a lower bound of the power-law behavior (in our case, *xmin* =3 was selected). However, empirical data scaling is commonly truncated at some upper scales. Indeed, the scaling behavior of the cumulative distributions in the control participants was visually broken over the scaling region > 30 min (Figure 2A). Thus, we fitted the distributions over the range of [3, 30] min using the Levenberg–Marquardt algorithm and compared the fitted scaling exponents using the Clauset method (Figure 2B). The estimated values were highly correlated (*r* = 0.97) in both groups, indicating the robustness of the estimated scaling exponents.

**TABLE 2:** Basic statistics and behavioral organization parameters of locomotor activity.

	Patients (*n* = 17)	Controls (*n* = 17)	*P*-value
Mean (count/min)	134.70 ± 20.83	143.85 ± 17.55	0.1756
SD (count/min)	113.72 ± 7.91	117.10 ± 6.10	0.1729
Resting period (min)	8.88 ± 1.75	8.36 ± 1.08	0.3054
Active period (min)	6.50 ± 1.63	6.66 ± 1.30	0.7458
γ	0.94 ± 0.13	1.02 ± 0.11	0.0496*
α	1.42 ± 0.11	1.48 ± 0.17	0.2171
β	0.55 ± 0.07	0.52 ± 0.11	0.3312

SD, standard deviation.

*Statistically significant difference (*p* < 0.05).

The cumulative distributions of active period durations in both groups showed a stretched exponential functional form (data not shown), but the estimated values of parameters α and β did not show any significant group difference. We also examined other statistical indices, mean activity level and standard deviations of locomotor activity, and mean resting and active periods, but did not find any significant group difference in those indices (Table 2).

### Factors potentially influencing parameter γ in delayed sleep phase disorder

A multiple linear stepwise regression analysis in patients with DSPD did not show a significant relationship between γ and patient’s clinical parameters: MEQ, BDI, remission or non-remission, social adjustment, sleepiness, or medicine (ramelteon, benzodiazepines or non-benzodiazepines, and vitamin B_12_). The final regression model constructed by a stepwise selection procedure included only social adjustment as a dependent variable, but it did not show statistical significance (*R*^2^ = 0.2146, *p* = 0.0611); the values of γ slightly decreased in patients with good social adjustment.

## Discussion

In this study, we demonstrated that the scaling exponent γ of the cumulative distributions of resting periods in DSPD was significantly lower than those in control participants, indicating a systematic and significant increase of durations of resting periods or lower activity levels in ultradian locomotor dynamics in patients with DSPD. This is consistent with our previous researches showing the decrease of γ in patients suffering from MDD (13, 14), implying the shared behavioral alterations between MDD and DSPD. There could be several explanations for this rest–activity pattern feature in DSPD patients. One of them is that DSPD may be a form of subclinical or prodromal depression. According to reports, DSPD is frequently accompanied by depression (1). The BDI score was slightly but significantly higher in patients with DSPD compared to control participants in this study, although the mean BDI score of patients with DSPD was in the range of “non-depressed” or “dysphoria” (25). Our patients had no obvious clinical depression and no comorbidity of other psychiatric disorders, and no patient enrolled in the study was taking antidepressant medications. Additionally, the multiple linear stepwise regression analysis did not exhibit significant associations of BDI scores with the values of γ in patients with DSPD. Nonetheless, it is plausible that DSPD has some continuity with MDD or similar psychopathology.

Some previous studies have used almost the same analysis for relevant conditions. In particular, it would be important to compare findings reported in chronic fatigue syndrome (CFS) because CFS has been reported to be associated with circadian disruption (26) and depression (27) as in DSPD. Kawabata et al. (28) studied the actigraphic rest–activity patterns of children with CFS, finding that resting periods tended to follow a power–law distribution in both children with CFS and healthy children without differing significantly between the groups. In contrast, when data for time out of bed (UP) and in bed (DOWN) were analyzed separately, the distributions did not always follow a power–law distribution. In children with CFS, UP tended to follow a power–law distribution and, interestingly, also showed a systematic increase in the incidence of longer resting periods compared with the incidence in healthy children. Kawabata el al. interpreted that these may be due to the instability of sleep in children with CFS. To some extent, the results reported by Kawabata et al. differ from ours because the distribution of resting periods in our patients with DSPD showed a systematic increase in the incidence of longer resting periods without separating UP and DOWN. That observation might potentially have been attributed to the difference in sleep (=DOWN) in patients with DSPD; however, sleep parameters and the distribution parameter γ showed no significant correlations (data not shown), and we consider that explanation to be unlikely. However, further studies, such as DOWN/UP periods analysis, would be necessary to more deeply understand the alteration in resting period durations in DSPD.

In two separate studies, Ochab et al. (29, 30) observed a systematically *decreased* incidence of longer resting periods (i.e., increased |γ|) after partial sleep deprivation in healthy adults. It is interesting that those changes were contradictory to those observed in DSPD and MDD, and partial sleep deprivation has been reported to relieve depression (31). As for our study, the amount of sleep can be seen to be non-significantly different between patients with DSPD and healthy participants (Table 1) and it is unlikely that some sleep deprivation would have impacted our results.

The shared characteristics of the rest–activity pattern between DSPD and MDD would be needed to be further explained. A simple explanation would be that psychomotor retardation would have resulted in a systematic increase in resting period durations. Our DSPD patients, on the other hand, do not always exhibit clinical psychomotor retardation in the narrow sense. We would like to propose another possible explanation for these characteristics: a shift in behavioral priority in decision-making processes. Barabasi (32) developed a stochastic priority based queuing model which can account for power-law distributions of waiting times (i.e., resting periods) observed in human social activities, such as communications, web browsing, and trade transactions, while whether the heavy tails observed in such activities follow power-law or not is still controversial (33). This model can theoretically explain the change in the cumulative distributions of resting periods (25, 26). In summary, the distribution of resting periods follows a power-law form when the highest-priority task is always chosen for execution among multiple ones, whereas the random selection of tasks results in an exponential distribution (25), and the stronger the prioritization in task selection, the fatter the tail of resting period distributions (i.e., decrease of the scaling exponent). It is possible that patients with MDD tended to respond “only” to higher physiological demands (14), which could result in a shift in the distribution of resting periods. Interestingly, we have observed in clinical practice that many patients with DSPD presented not only circadian sleep disturbances but also some behavioral alterations. The patients appear to exhibit “slower-paced” activity levels in daily life; for example, they cannot promptly start their tasks (e.g., homework, daily routines, and even habitual behaviors such as going to bed at a scheduled time). Further, they are often not punctual (e.g., work deadlines, doctor appointments), and they sluggishly continue and/or cannot stop their “easy-going” activities (e.g., mobile games, internet browsing). In contrast, they sometimes spring into action when faced with critical situations (e.g., important examination, crisis of losing their job). These behavioral characteristics of DSPD differ from those of classical psychomotor retardation, and in our clinical experience, they can be observed without the presence of other psychiatric conditions such as MDD. We believe these clinical observations could be also explained by the same scenario based on the stochastic priority model. This may also be consistent with recent findings that a significant proportion of patients with DSPD may have behavioral components in their etiology (3).

It is interesting to note that mice with the mutation of a circadian clock gene, *Period2*, exhibit the decrease of the scaling exponent γ of resting period distributions in their activities (13). There are many reports suggesting the interrelations between (1) mood disorder and circadian sleep disruptions (6), (2) mood disorder and clock gene mechanisms (11, 12), and (3) DSPD and clock genes (2, 34). The similarity in behavioral changes between *Period2* mutant mice and patients with DSPD may support the relationship between the development of DSPD and dysfunctions of chronobiological mechanisms, including circadian clock genes. It is also interesting that clock genes, including *Period2*, are possibly associated with reward systems (35, 36), suggesting that the circadian system may have a complicated association with fundamental behavioral mechanisms, especially in these disorders, rather than simply determining the circadian phase. Of course, the results of the present study do not support further discussion of those mechanisms; more research will be required.

We need to interpret our results with caution. Other factors likely to affect our results might be medication, diurnal sleepiness, and social adjustments. First, 15 out of 17 patients with DSPD took psychotropic or circadian-modulating medicines, whereas, all control participants did not. Although the patients did not take antipsychotic or antidepressants with a sedative effect, they were medicated with ramelteon, benzodiazepines or non-benzodiazepines, and vitamin B_12._ Benzodiazepines and non-benzodiazepines are known to have more or less sedative effects (37), but most of them are short-acting sleep medicines. Therefore, we believe the effect of these medications on locomotor activity during daytime was limited. Ramelteon and vitamin B_12_ might have some effect on circadian rhythm (38, 39), but their effect on micro-fluctuations in locomotor activity at times-scales of ultradian rhythms are unclear. Considering the lack of any significant association between the use of those medicines and γ in the multiple linear regression analysis, their apparent effects were probably not real.

Second, one may consider the influence of diurnal sleepiness. In general, patients with DSPD are unable to keep their own internal biological rhythm entrained to social schedules or environmental light–dark cycles. Therefore, they often become sleepy and are likely to take naps during the day. The frequent occurrence of short daytime naps may have resulted in lowered values of parameter γ, if some mathematical requirements, such as power-law behaviors in nap durations, are satisfied. In this study, however, there was no association between sleepiness and parameter γ. In addition, patients did not report naps on their sleep logs nor were any naps apparent in actigraphy during the recording period. Therefore, sleepiness would have no direct influence on parameter γ.

A third factor of influence might be social adjustment. The physical activity levels in patients who cannot adjust to social activity (e.g., school or work) are expected to decrease because the occurrence of longer resting periods in diurnal locomotor activity data is thought to increase. But in regression analysis, there was statistically marginal (*p* = 0.0611) association between social adjustment and parameter γ with positive slope, indicating that patients with good social adjustment tend to show lower values of γ, and the absence of the contribution of social adjustment increase with the durations of resting periods/lower activities. Although it is insignificant, these contrary results should be interpreted carefully.

There are several limitations of this study. First, the small sample size might be problematic because it directly affects the statistical power to detect significance, which might limit our ability to reveal significant associations with clinical factors. In addition, the possibility that the statistical significance in γ due to a type 1 error cannot be eliminated. Second, to rigorously prove our hypothesis, various clinical factors discussed above should be controlled in the study. For example, the study protocols under drug-free and/or off-duty conditions should be considered. In addition, the assessment of sleepiness using the Epworth Sleepiness Scale (40) or circadian phase based on dim light melatonin onset (41) would provide useful information. Such well-controlled studies with a larger sample sizes are essential to generalize our findings. Third, we did not objectively or quantitatively evaluate the behavioral characteristics in the DSPD patients; this could help validate our hypothesis and stimulate further discussion.

In conclusion, we found that the value of the scaling exponent γ of cumulative distributions of resting periods in locomotor activity at time scales of ultradian rhythms significantly decreased in patients with DSPD, which was similar to those in patients with MDD. The shared alteration in the behavioral organization suggested the existence of similar pathophysiology between DSPD and MDD, possibly related to shared strategic changes in selecting and initiating daily tasks/work. We believe such changes result in the characteristic behaviors clinically observed in patients with DSPD.

## Data availability statement

The raw data supporting the conclusions of this article will be made available by the authors, without undue reservation.

## Ethics statement

This study was approved by the Ethics Committee of the Fujita Health University and Department of Sleep Medicine, Toyohashi Mates Sleep Center. Written informed consent to participate in this study was provided by the participants, and in a case of minor, by the participant and his/her one parent.

## Author contributions

MH and TK contributed to study design, data collection, data analysis, interpretation of results, and preparation of the manuscript. TN and YY contributed to study design, data analysis, interpretation of results, and preparation of the manuscript. AW and YE contributed to study design, data collection, data analysis, and interpretation of results. SK contributed to data collection and preparation of the manuscript. NI contributed to study design and preparation of the manuscript. All authors read and approved the final manuscript.
