# Supplementary material for: Altered distribution of resting periods of daily locomotor activity in patients with delayed sleep phase disorder
Source: Front Psychiatry. 2022 Oct 13;13:933690. doi: 10.3389/fpsyt.2022.933690 (PMC9606617; doi:10.3389/fpsyt.2022.933690)
Supplement: Supplementary file 1 [file Table_1.docx]

Table S1. The list of the references in Japanese.

21. 北島剛司, 目片隆宏, 服部美穂, 奥田明子, 赤松拡, 金森亜矢, 宅野智子, 岩田仲生. 睡眠覚醒リズム障害の症状評価尺度作成の試み. 不眠研究*2007* (2007):17-24.

22. 中村亨, 菊池裕絵, 吉内一造, 山本義春. 新たな臨床・研究発展にむけて-異分野との連携を探る-数理科学モデルから精神行動異常を解く. 精神科 (2011)**18**:554-559.
